# Targeting Spleen Tyrosine Kinase-Bruton's Tyrosine Kinase Axis for Immunologically Mediated Glomerulonephritis

**DOI:** 10.1155/2014/814869

**Published:** 2014-03-30

**Authors:** Jin-Shuen Chen, Li-Chien Chang, Shyh-Jer Huang, Chao-Wen Cheng

**Affiliations:** ^1^Division of Nephrology, Department of Internal Medicine, Tri-Service General Hospital, Taipei 114, Taiwan; ^2^School of Pharmacy, National Defense Medical Center, Taipei 114, Taiwan; ^3^School of Pharmacy, College of Pharmacy, China Medical University, Taichung 404, Taiwan; ^4^Chinese Medicine Research and Development Center, and Center for Molecular Medicine, China Medical University Hospital, Taichung 404, Taiwan; ^5^Graduate Institute of Clinical Medicine, College of Medicine, Taipei Medical University, Taipei 110, Taiwan; ^6^Graduate Institute of Medical Sciences, National Defense Medical Center, Taipei 114, Taiwan

## Abstract

The importance of B-cell activation and immune complex-mediated Fc-receptor activation in the pathogenesis of immunologically mediated glomerulonephritis has long been recognized. The two nonreceptor tyrosine kinases, spleen tyrosine kinase (Syk) and Bruton's tyrosine kinase (Btk), are primarily expressed by hematopoietic cells, and participate in B-cell-receptor- and Fc-receptor-mediated activation. Pharmacological inhibitors of Syk or Btk are undergoing preclinical development and clinical trials for several immune diseases; and Syk inhibitors have been shown to reduce disease activity in rheumatoid arthritis patients. However, the clinical therapeutic efficacies of these inhibitors in glomerulonephritis have not been evaluated. Herein, we review recent studies of Syk and Btk inhibitors in several experimental primary and secondary glomerulonephritis models. These inhibitors suppressed development of glomerular injury, and also ameliorated established kidney disease. Thus, targeting Syk and Btk signaling pathways is a potential therapeutic strategy for glomerulonephritis, and further evaluation is recommended.

## 1. Immunologically Mediated Glomerulonephritis

Although inflammatory components might not necessarily be involved, the formation of immune deposits at various intraglomerular locations occurs with most forms of glomerulonephritis. Immunoglobulin A (IgA) nephropathy, lupus nephritis, and postinfectious glomerulonephritis are the most common forms of immune-dependent glomerulonephritis. Immune deposits may form from mechanisms of either* in situ* immune-complex formation or by the trapping of circulating immune-complexes. In primary glomerulonephritis, an antibody can specifically bind to intrinsic antigens in normal glomerular structures or to nonspecific localized soluble antigens in glomeruli. These immune-complexes may be deposited on subepithelial, subendothelial, and mesangial regions, and the clinical and morphological features are mainly determined by the location of immune deposits and the targeted glomerular cell types. Due to special physical and anatomical features, the kidney is also more susceptible to circulating immune-complex deposition, which causes secondary glomerulonephritis. Therefore, activation of B cells is an early event in the initial stage of these diseases; consequently, they mature into antibody-producing plasma cells that express antibodies, target specific antigens, and form immune-complexes. Once immune-complexes are deposited in glomeruli, the Fc portion of immunoglobulins in immune-complexes binds to Fc receptors on effector cells of the immune system and kidney [1]. This engagement transduces activating signal pathways such as phospholipase C-(PLC-)*γ* and phosphatidylinositol-3 kinase (PI3 K) [2] and triggers activation of intrinsic glomerular cells or infiltrating leukocytes to release many inflammatory mediators, such as complements, vasoactive substances, cytokines, and coagulation factors [1, 3, 4]. The processes of immune-complex formation and binding to the Fc receptor might both be important therapeutic targets for glomerulonephritis. To date, treatment has been practically limited to immunosuppression with cyclophosphamide or azathioprine and, in the last decade, the use of mycophenolate mofetil, all in combination with nontargeted high-dose glucocorticoids [5]. Combined regimens with mycophenolate mofetil can relieve treatment-related cytotoxicity and present comparable efficacies of inducing remission and maintenance therapy; however, high-dose steroids are still a necessary adjunct treatment. It was also reported that long-term continuous treatment with corticosteroids and mycophenolate mofetil as both initial and maintenance immunosuppression for severe proliferative lupus nephritis resulted in relatively favorable renal and patient outcomes in Chinese lupus nephritis patients [6]. According to a European cohort study, over 50% of lupus nephritis patients still needed immunosuppressive therapy for 10 years after a diagnosis [7]. Even though the therapeutic effects of long-term steroid treatment are favorable, many side effects are associated with their use [8]. New therapeutic experimental approaches and targeted therapeutic regimens are needed to improve the management of glomerulonephritis.

## 2. Immunological Regulation by the Spleen Tyrosine Kinase (Syk-)Bruton's Tyrosine Kinase (Btk) Axis

Syk is a cytoplasmic nonreceptor tyrosine kinase that has an important role in receptor signaling in hematopoietic cells including B cells, neutrophils, monocytes/macrophages, and T cells. It plays a critical role in intracellular signal transduction of classical immunoreceptors associated with immunoreceptor tyrosine-based activation motifs (ITAMs), including the B-cell receptor (BcR) and Fc receptor (FcR). In addition to hematopoietic cells, Syk is also expressed by nonhematopoietic cells, such as fibroblasts, mammary epithelial cells, hepatocytes, synoviocytes, and certain solid tumor cells. In these cell types, activation of Syk appears to be mediated through an ITAM-independent pathway by multiple stimuli, including interleukin-1 (IL-1), integrin, lipopolysaccharide, and tumor necrosis factor- (TNF-) *α* [9], though the underlying mechanisms are currently unknown. The roles of the Syk-Btk axis in innate immune cell function and tumor cell progression were critically reviewed [10]. In the BcR and FcR signaling pathway, engagement of BcR and FcR activates receptor-bound Src family protein-tyrosine kinases, such as Lyn, Blk, and Fyn, and phosphorylates tyrosine residues in receptors of ITAMs. Tyrosine-phosphorylated ITAMs then recruit Src family members and Syk kinases via the binding domain of phosphotyrosine-binding Src homology 2 and regulate conformational change-dependent Syk activation. Activated Syk kinase can affect the phosphorylation of Btk, cooperatively regulate activation of PLC-*γ*, and generate diacylglycerol (DAG) and inositol 1,4,5-trisphosphate (IP3) which affect calcium mobilization. Consequently, DAG and calcium promote activation of protein kinase C (PKC) and activate mitogen-activated protein kinase (MAPK) family (extracellular signal-regulated kinase (ERK), c-Jun N-terminal kinase (JNK), and p38) downstream signaling cascades [10]. Activation of transcription factors, such as activator protein-1 (AP-1), nuclear factor of activated T cells (NFAT), and nuclear factor- (NF-) *κ*B, leads to alterations in multiple cellular activities, including proliferation, differentiation, inflammatory mediator release, and phagocytosis.

Although mice deficient in Syk and Btk showed some different phenotypic features, deficiencies in either one of the kinases showed profound hematopoietic defects and reduced the susceptibility to certain experimental autoimmune disease models, such as systemic lupus erythematosus (SLE) and rheumatoid arthritis (RA). Syk-deficient B-lineage cells in the bone marrow were arrested at the pro-B-cell stage, while pre-B cells and immature B cells exhibited reduced maturation and survival [11]. Macrophages derived from Syk-deficient mice were defective in FcR-mediated phagocytosis and had diminished FcR-induced MAPK activation [12]. Mutations in the Btk gene in humans were reported to cause inherited immunodeficiency and X-linked agammaglobulinemia, which features the absence of peripheral B cells, reductions in all classes of serum immunoglobulins, and a less-severe phenotype in mice with an X-linked immunodeficiency (*xid*). Macrophages of* xid* mice had impaired functions in generating reactive oxygen intermediates and proinflammatory cytokines [13]. Moreover, cultured Btk-deficient mast cells revealed defects in degranulation and cytokine production upon Fc*ε*R stimulation [14]. Therefore, disruption of the Syk-Btk axis may reduce both BcR- and FcR-mediated immune responses and thus lessen the severity of immunological diseases.

In recent findings, a Syk kinase inhibitor (fostamatinib, Rigel Pharmaceuticals) was reported to produce beneficial outcomes in RA clinical trials [15, 16]. In addition, Syk inhibitors also presented inhibition abilities in FcR-mediated activation of monocytes/macrophages, basophils and neutrophils, and BCR-mediated activation of B lymphocytes [17, 18]. The Btk inhibitor, ibrutinib (Imbruvica, Pharmacyclics), was approved by the US FDA in November 2013 for treating mantle cell lymphomas [19] and presented encouraging clinical efficacy and tolerability profiles in patients with relapsed chronic lymphocytic leukemia/small lymphocytic lymphomas [20]. With the promising therapeutic potential for targeting immunological disorders and human malignancies by suppressing Syk and Btk, a number of compounds are still undergoing preclinical development and clinical trials [10, 21]. In this minireview, we focus on recent findings of potential therapeutic applications of targeting the Syk-Btk axis in experimental glomerulonephritis models (Tables 1 and 2).

## 3. Targeting the Syk-Btk Axis for Treatment of Glomerulonephritis

### 3.1. Lupus Nephritis

Lupus nephritis is a severe and frequent organ complication in SLE-affected patients. The main pathological feature of SLE is the overproduction of circulating antinuclear proteins and anti-DNA autoantibodies. After binding to self-antigens, circulating immune-complexes are formed that deposit on cell surfaces, and deposited immune-complexes induce inflammatory cascades that lead to tissue injury. Therefore, therapeutic strategies may be theoretically achieved by both diminishing B-cell activation and suppressing FcR-mediated immune responses. Indeed, reduced B-cell activity and depleted numbers of B cells were successfully used to treat lupus nephritis, RA, and antineutrophil cytoplasmic antibody-associated vasculitis [30–34]. Targeting BcR activation and FcR responses by Syk and Btk inhibitors may provide alternative therapeutic regimens for lupus nephritis.

Fostamatinib, an oral prodrug of the selective Syk inhibitor R406, showed efficacy in severe nephritis in two lupus nephritis animal models. Bahjat et al. showed that treatment with fostamatinib either prior to or after the onset of disease delayed the progression of proteinuria and azotemia, reduced the renal injury, and prolonged the survival in lupus-prone NZB/NZW mice [22]. In another study, Deng et al. evaluated the therapeutic effects of fostamatinib in renal and skin lesions in an MRL/lpr lupus-prone model. Fostamatinib treatment showed both preventive and therapeutic effects in renal and skin diseases. Sustained benefits from fostamatinib resulted in extended suppression for a couple of weeks after drug cessation [23]. It should be noted that even though fostamatinib treatment reduced lymphadenopathy and frequencies of splenic B-cell and T-cell subpopulations, the serum anti-dsDNA autoantibody titers were less affected, while glomerular depositions of IgG, IgM, and C3 were all reduced in fostamatinib-treated mice [22, 23]. These findings represent similar results to FcR*γ*-deficient NZB/NZW mice which generated and deposited immune-complexes and activated complements but were protected from severe nephritis [35]. Indeed, fostamatinib treatment also showed similar results to CD16/CD32 FcR-blocking antibody treatment in reducing Arthus responses [22]. The therapeutic efficacy of fostamatinib in these lupus mice models may have mainly resulted from immune-complex downstream FcR-dependent mechanisms.

It was reported that a mutation of the Btk gene abrogated autoimmune disease exhibited in Lyn-deficient mice by reducing peripheral mature B cells and serum IgM and IgG3 levels [36]. In addition, aged CD19-hBtk and MHCII-hBtk transgenic mice showed spontaneous autoimmune features characterized by increasing serum antinucleosome and anti-dsDNA autoantibodies, glomerular deposition of IgG1, IgG2c, and IgG3, glomerular hypercellularity, and mesangial matrix expansion [37]. Several Btk inhibitors were evaluated in lupus nephritis models. Ibrutinib treatment prior to disease onset in B6.Sle1/B6.Sle1.Sle3 mice decreased renal damage and reduced levels of circulating antinucleosome, antihistone, and anti-ssDNA autoantibodies [27]. Both RN486 and PF-06250112 showed reduced severity of established glomerulonephritis in NZB/NZW mice. Treatment with these Btk inhibitors prevented similar features in the development of proteinuria and reduced production of inflammatory cytokines, inflammatory infiltrates, and glomerulosclerosis. In addition, B-cell activation, circulating autoantibody levels, and glomerular immune-complex deposition were all inhibited [28, 29].

### 3.2. Nephrotoxic Serum Nephritis (NTN)

NTN is a rapid-onset immune-complex- and FcR-dependent disease model, which is induced by an intravenous injection of exogenous antiglomerular basement membrane (anti-GBM) antibodies. This model resembles Goodpasture's syndrome in humans and is characterized by the formation of crescents and IgG and C3 linear depositions along the GBM. In this model, preventive treatment with fostamatinib inhibited the development of proteinuria and glomerular fibrinoid necrosis and reduced inflammatory cell infiltrates. In addition, treatment after the onset of nephritis also decreased the severity of glomerular injury, especially manifested in the diminished formation of glomerular crescents and reduced renal monocyte chemoattractant protein-1 (MCP-1) and IL-1*β* production [24]. Another Syk inhibitor from Celgene Corporation also showed protective effects via reducing glomerular JNK and p38 MAPK activation and resulted in protection from proteinuria and glomerular thrombosis and reductions in glomerular messenger (m)RNA levels of proinflammatory molecules and acute glomerular neutrophil influx [25]. Interestingly, fostamatinib failed to reduce exogenous glomerular IgG deposition but showed inhibition of endogenous glomerular IgG deposition with preventive treatment [24]. This suggests that fostamatinib targets immune-complexes downstream of FcR-dependent signaling and inhibits proinflammatory cytokine and chemokine production and inflammatory infiltrates.

In addition, serum levels of an antiexogenous IgG antibody were suppressed with preventive treatment in animals; this may represent evidence that early BcR activation was also inhibited by fostamatinib. However, it failed to reduce exogenous and endogenous glomerular IgG deposition with established nephritis [24]. These results suggest that fostamatinib may inhibit early BcR-mediated activation but fails to suppress constitutively activated antibody-producing plasma cells. This inference may explain the sustained serum anti-dsDNA autoantibody titers and glomerular immune-complex deposition in fostamatinib-treated lupus nephritis.

The therapeutic efficacy of the Btk inhibitor, PF-06250112, in a passive model of NTN was also reported [29]. The model was induced by passive transfer of both a rabbit-derived anti-murine GBM antibody and a murine anti-rabbit antibody. Mice treated with PF-06250112 exhibited a dose-dependent decrease in proteinuria levels, but it did not affect the intensity of glomerular IgG and C3 deposition. This finding indicates that the Btk inhibitor, PF-06250112, showed similar effects to the Syk inhibitor, fostamatinib, in FcR-dependent glomerulonephritis.

### 3.3. IgA Nephropathy (IgAN)

IgAN is the most common type of glomerulonephritis that features granular mesangial IgA deposits. Glomerular deposition of circulating IgA immune-complexes is considered the primary pathogenic mechanism [38]. Deposited IgA immune complexes trapped by Fc*α*RI on mesangial cells induced ITAM phosphorylation and Syk and Btk activation, elicited mesangial cell proliferation and proinflammatory and profibrogenic mediator release, and led to the progression of IgAN [39]. From immunohistochemical staining in IgAN clinical cases, both total and phospho-Syk were increased in glomeruli and glomerular tufts and crescents. Treatment with either the pharmacological Syk inhibitor, R406, or Syk siRNA reduced pathogenic IgA1-induced MCP-1, IL-6, IL-8, interferon- (IFN-)*γ*, IP-10, RANTES, and PDGF-BB production in human mesangial cells. In addition, cell proliferation was also inhibited [26]. Syk activation participates in the downstream signaling of IgAN and may serve as a therapeutic target. The role of Btk has not been explored in IgAN and thus the potential therapeutic efficacy of a Btk inhibitor requires an* in vivo* study for further investigation.

## 4. Summary and Conclusions

Current therapies for glomerulonephritis rely on nonspecific immunotherapy and are mostly associated with long-term side effects. It is known that dysregulation of multiple immune cells contributes to the pathogenesis of glomerulonephritis; among these, activation of B-cell- and immune-complex-mediated inflammation is a hallmark of the disease. Several therapeutic strategies presented beneficial outcomes by specifically targeting these pathways of glomerulonephritis, such as rituximab for B-cell depletion, belimumab for blocking the B-lymphocyte stimulator receptor, and eculizumab for complement inhibition. Since Syk and Btk showed important regulation of both BcR and FcR signaling, specific pharmacological inhibitors for these kinases are currently undergoing preclinical and clinical trials as treatment for autoimmune diseases and hematological cancers. However, there is still a lack of data from clinical trials of Syk and Btk inhibitors in glomerulonephritis (fostamatinib, NCT00752999; the SOLEIL study was withdrawn prior to enrollment). According to* in vitro* and animal models of primary and secondary glomerulonephritis as described above, both Syk and Btk inhibitors produced improvements in glomerular injury, especially in attenuating FcR-mediated inflammation, such as inflammatory cytokines and inflammation leukocyte recruitment. A Btk inhibitor showed superior efficacy to a Syk inhibitor in diminishing autoantibody production, which implies relief of B-cell activation. It is of note that some of the Btk inhibitors are irreversible inhibitors; the assessment of different types of Btk inhibitors in glomerulonephritis still needs further consideration. Both Syk and Btk have considerable potential as therapeutic targets for glomerulonephritis, but future studies of clinical evaluations of their inhibitors and combination regimens are still required.

## Figures and Tables

**Table 1:** Effects of spleen tyrosine kinase (Syk) inhibitors on glomerulonephritis.

Clinical disease	Model	Treatment	Results	Reference
Lupus nephritis	NZB/W	Fostamatinib	*As prophylactic treatment * Delayed proteinuria and azotemia Reduced renal pathology and prolonged survival Did not affect serum anti-DNA autoantibodies *In full-blown disease * Decreased the incidence and severity of renal pathology	[22]
	MRL/lpr	Fostamatinib	*As prophylactic treatment * Prevented development of proteinuria and suppressed pathologic changes *In full-blown disease * Improved proteinuria Decreased lymphadenopathy Did not affect serum anti-DNA autoantibodies	[23]

Antiglomerular basement membrane nephritis	NTN	Fostamatinib	*As prophylactic treatment * Improved proteinuria Reduced glomerular fibrinoid necrosis and infiltration of inflammatory cells Increased exogenous antibody deposition in glomeruli Suppressed levels of both circulating and glomerulus-deposited endogenous antibodies *In full-blown disease * Improved proteinuria and serum creatinine levels Increased exogenous antibody deposition in glomeruli Reduced renal MCP-1 and IL-1*β* production	[24]
	NTN	SYK inhibitor (Celgene Corp.)	*As prophylactic treatment * Prevented proteinuria, thrombosis, and platelet activation Inhibited JNK and p38 MAPK activation Reduced glomerular inflammation leukocyte recruitment	[25]

IgA nephropathy	*In vitro *	R406; siRNA	*In vitro study* Decreased heat-aggregated IgA1-induced IL-6, IL-8, IFN-*γ*, IP-10, RANTES, and PDGF-BB production by human mesangial cells Inhibited heat-aggregated IgA1-induced human mesangial cell proliferation	[26]

**Table 2:** Effects of Bruton's tyrosine kinase (Btk) inhibitors on glomerulonephritis.

Clinical disease	Model	Treatment	Results	Reference
Lupus nephritis	B6.Sle1 B6.Sle1.Sle3	PCI-32765	*As prophylactic treatment * Decreased renal damage and lymphocyte infiltration Reduced levels of antinucleosome, antihistone, and ssDNA autoantibodies	[27]
	NZB/W	RN486	*In full-blown disease * Improved proteinuria Reduced glomerulosclerosis and immune-complex deposition Inhibited B-cell activation and anti-dsDNA autoantibody secretion Downregulated expressions of inflammatory cytokines	[28]
	NZB/W	PF-06250112	*In full-blown disease * Prevented the development of proteinuria Reduced glomerular injury and inflammatory infiltrates Significantly reduced both IgG and C3 depositions	[29]

Antiglomerular basement membrane nephritis	NTN	PF-06250112	*As prophylactic treatment * Dose-dependently decreased proteinuria levels Did not affect either IgG or C3 deposition	[29]
